# Diuretic-associated admissions to the emergency department – too hot to handle? Results from the ADRED-study

**DOI:** 10.1186/s12916-026-05370-1

**Published:** 2026-11-07

**Authors:** Jakob Sommer, Justyna Wozniak, David Wißmann, Judith Berres, Verena Graeff, Andrea Kriegisch-Stumpf, Anja Knüppel-Ruppert, Harald Dormann, Julia C Stingl, Katja S Just

**Affiliations:** 1 Department of Clinical Pharmacology and Pharmacoepidemiology, Medical Faculty Heidelberg / Heidelberg University Hospital, Heidelberg University, Heidelberg, Germany; 2 Institute of Clinical Pharmacology, University Hospital RWTH Aachen, Aachen, Germany; 3 Central Emergency Department, University Hospital Augsburg, Augsburg, Germany; 4 Central Emergency Department, Hospital Ingolstadt, Ingolstadt, Germany; 5 Central Emergency Department, Hospital Fürth, Fürth, Germany; 6 Department of Interprofessional Hospital Development, University Hospital Augsburg, Augsburg, Germany; 7 Pharmacology I, Faculty of Medicine and University Hospital Cologne, University of Cologne, Cologne, Germany

**Keywords:** Diuretics, Adverse drug reaction, Climate, Heat, Emergency department, Segmented regression

## Abstract

**Background:**

Rising ambient temperatures and increasing thermal stress are major public health challenges, associated with increased emergency department (ED) visits in Europe. Frequently prescribed diuretics may exacerbate heat-related risks through mechanisms such as dehydration and electrolyte imbalance. Although many studies have shown a connection between temperature and mortality, the effect of cumulative ambient temperature on diuretic-associated adverse drug reactions (ADRs) remains underexplored. This study investigates whether higher temperatures are associated with an increase in diuretic-related ADRs in ED admissions.

**Methods:**

Data from a prospective, observational study on ADRs leading to ED admission (ADRED-study) with a standardized causality assessment of documented drugs were analyzed. Linear, polynomial, and segmented regression were applied to quantify the relationship between 5-day mean temperature and the ratio of suspected diuretic-related ADRs to all diuretic uses.

**Results:**

A significant relationship between higher 5-day mean temperatures and more diuretic-associated ADRs was found in linear regression analysis (β = 0·0035, 95%CI: 0·0015 − 0·0054, R²=0·188). A segmented regression model identified a critical threshold of ~ 15·7 °C (adjusted R^2^ = 0·382). After the threshold at higher temperatures, the slope for diuretic-associated ADRs increased significantly (β = 0·0201, 95%CI: 0·0068–0·0333). The spline model similarly showed a high statistical fit (adjusted R^2^ = 0·399), indicating a non-linear, accelerating increase in ADRs at higher temperatures.

**Conclusions:**

Diuretic-associated ADRs increase non-linearly with ambient temperature, with a breakpoint at approximately 15·7 °C. Especially diuretic-medicated individuals may be vulnerable to cumulative thermal stress.

**Trial registration:**

The ADRED-study was registered in the German Clinical Trials Register under DRKS00008979.

**Supplementary Information:**

The online version contains supplementary material available at https://doi.org/10.1186/s12916-026-05370-1.

## Background

In the last years, the consequences of climate change on health have received increased attention [1]. The 2024 Europe report of the Lancet Countdown on health and climate change showed the negative impact of climate change on health in Europe. In the last decade, the number of person-days of heatwave exposure increased by 97% compared to the decade before [2]. Along with heatwaves, more patients with renal diseases need medical care, especially affecting older adults [3]. Consequently, there were at least 60,000 heat-related deaths in Europe in summer 2022 [4] with an observed higher risk for women compared to men [2, 4].

Many older adults are generally multi-medicated, while female sex is often associated with an even higher number of drug intake [5]. Drugs can add to the heat-related risk; for example the use of vasoactive drugs being associated with mortality in the context of heatstroke [6]. Adverse drug reactions (ADRs) are estimated to cause around 6.5% of admissions to the emergency department (ED) [7, 8] with the majority being dose-associated ADRs [8]. Several mechanisms may link diuretic use to heat-related morbidity and mortality, including increased diuresis, electrolyte imbalance, altered thermoregulation, and reduced thirst recognition [9]. The use of diuretics is associated with heatstroke-related mortality [10] and during higher temperatures, increased prevalence of diuretic-associated hyponatremia can be observed with again higher rates in females [11].

The aim of this study was to analyze if more diuretic-associated ADRs can be seen with higher temperatures in ED admissions.

## Methods

### Study population

Data of the ADRED-study (Adverse Drug Reactions in Emergency Departments; trial registration DRKS-ID: DRKS00008979) was analyzed. The ADRED-study was a multicenter, prospective, observational study collecting ADR cases of adults in six EDs of tertiary care and academic teaching hospitals in Germany. Cases were enrolled after standardized causality assessment by trained study personnel between 2015 and 2021 [12]. Details on the study design are published elsewhere [7, 13]. The ADRED-study was approved by the responsible ethical committee of the University of Bonn (202/15).

### Data compilation

Weather data from the closest available German Meteorological Service *(Deutscher Wetterdienst)* station to each recruiting ED were used and matched to the dates of ED admission [14]. These stations were located within a  < 20 km straight-line distance from the respective study centers. For each included case, the corresponding temperature data measured at two meters above ground were assigned from the nearest station.

The weather data included daily maximum temperature, daily mean temperature (in °C), and daily precipitation (in mm) at the respective station. While the primary analysis utilized 5-day mean values based on the date of admission and the four preceding days, additional windows (1-, 3-, and 7-day lags) were calculated for sensitivity analysis. All drugs documented upon ED admission were classified using the Anatomical Therapeutic Chemical (ATC) classification system [15]. Diuretics were defined using ATC-codes and grouped according to their suggested relation to the ADR causing ED admission – classified as diuretic-associated (possible, probable, or certain causality according to the WHO-UMC causality assessment), or not [12]. A table defining the use of diuretics by ATC-codes can be found in supplement 1. The ADRED-study dataset was filtered for patient cases with diuretic use.

Diseases previously diagnosed before ED admission, which could be associated with diuretic use, were defined using ICD-10 codes: acute kidney injury (AKI, N17), chronic kidney disease (CKD, N18), heart failure (I50), and essential hypertension (I10). In addition, we assessed the five most frequent admission diagnoses in patients with diuretic-associated ADRs using ICD-10 codes. To enable a control group, beta-blocker-associated ADRs were likewise analyzed. To this end, beta-blockers were defined as ATC code of C07.

### Statistical analysis

The preparation of the data set and the descriptive analyses were performed using Python 3·10·18, pandas 2·2·3, scikit-learn 1·6·1· and NumPy 2·0·1·.

To enable a robust assessment of the association between ambient temperature and diuretic-associated ADRs despite heterogeneous recruitment periods, study locations, and non-longitudinal patient observations, cases were grouped by temperature rounded to the nearest 0.5 °C. Groups containing fewer than 20 cases were merged with the next higher temperature group to ensure adequate sample size and to generate robust ratios. If the highest temperature group contained fewer than 20 cases, it was merged with the preceding lower temperature group, instead. In each case, the average temperature of the merged group was used, and the means for age and sex were calculated. Environmental and temporal covariates were similarly accumulated: 5-day mean precipitation was averaged across the entire temperature group, while the day of the week was summarized by calculating the total count of occurrences for each weekday within the group. For each temperature group, the ratio of the suspected drug class to all users of that drug class was calculated (diuretics in the primary analysis, sD/aD-ratio; beta-blockers in the comparative analysis, sB/aB-ratio). We removed all cases with concomitant diuretics intake from the beta-blocker comparative group. Grouping by temperature generated comparable, size-normalized estimates and allowed us to compare the relative frequency of suspected drug-related ADRs across temperature ranges while accounting for differences in the number of patients observed at each temperature level. These preparation steps were applied identically to both the diuretic and beta-blocker datasets to ensure comparability in the subsequent modeling.

The statistical analyses were made using R 4·5·0 with the packages tidyverse 2·0·0, segmented 2·1, splines 4·5·1, and ggplot2 3·5·2. Multivariate models were fitted for both drug classes. Initially, a linear regression model used 5-day mean temperature as the independent variable, adjusted for age, female sex, and mean 5-day precipitation.

To explore potential non-linear and accelerating relationships, we fitted a third-degree polynomial regression model, incorporating linear, quadratic, and cubic terms, as well as a natural cubic spline model (df = 3). The spline model was specifically utilized to capture complex curvature and potential tipping points without the constraints of global polynomial terms.

Additionally, we aimed to identify a temperature cut-off at which the risk of diuretic-associated ADRs increases significantly more strongly. Thus, a segmented linear regression model was fitted using the “segmented” package in R. Potential structural breaks in the relationship between temperature and sD/aD- and sB/aB-ratio were assessed using the Davies’ test.

Model fit was evaluated using R^2^ and similar model performances were further compared using AIC (Akaike Information Criterion). Sensitivity analyses were conducted by comparing models across various time lags (1-day to 7-day windows) and temperature metrics (mean vs. maximum temperatures) to justify the selection of the 5-day mean temperature. The model plots were created using ggplot2.

## Results

A total of *N* = 3601 patient cases with documented diuretic use were filtered from the ADRED-study with 31·4% (*n* = 1131) being assessed as diuretic-related ADRs.

Patients with diuretic-associated ADRs included a significantly higher proportion of females (51·9%, *n* = 587 vs. 46·1%, *n* = 1139) and showed a higher prevalence of pre-existing conditions, particularly kidney diseases and heart failure. Acute kidney injury was present in 17·3% (*n* = 196) vs. 8·7% (*n* = 215), and chronic kidney disease in 31·8% (*n* = 360) vs. 25·3% (*n* = 625). Heart failure was more frequent as well (25·8%, *n* = 292 vs. 22·1%, *n* = 547) compared to patients with ADRs unrelated to diuretics.

The five most frequent admission diagnoses in patients with diuretic-associated ADRs such as AKI, syncope, heart failure, volume deficit, and electrolyte disorders were significantly more common in these cases than in other non-diuretic-related ADRs. The control group was more urgently triaged compared to the group with diuretic-associated ADRs (Table 1).

**Table 1:** Description of the diuretic-treated cohort (*N* = 3601) stratified to patients with and without diuretic-associated adverse drug reactions

	Patients without suspected diuretics, *n* = 2470 (68·6%)	Patients with suspected diuretics, *n* = 1131 (31·4%)	*p*-value
Age (in years), median (IQR)	78 (70;83)	79 (71;84)	0·058
Female, n (%)	1139, 46·1%	587, 51·9%	0·001
Number of drugs, median (IQR)	10 (7;12)	9 (6;12)	< 0·001
Number of diuretics per patient, median (IQR)	1 (1; 2)	1 (1; 2)	< 0·001
Number of pre-diagnosed (PD) diseases, median (IQR)	8 (5;12)	9 (5;13)	< 0·001
PD N17 AKI, n (%)	215, 8·7%	196, 17·3%	< 0·001
PD N18 CKD, n (%)	625, 25·3%	360, 31·8%	< 0·001
PD I50 Heart failure, n (%)	547, 22·1%	292, 25·8%	0·017
PD I10 Essential hypertension, n (%)	1585, 64·2%	717, 63·4%	0·680
Number of admission diagnoses, Median (IQR)	1 (1;2)	1 (1;2)	0·379
N17 AKI, n (%)	77, 3·1%	148, 13·1%	< 0·001
R55 Syncope, n (%)	56, 2·3%	70, 6·2%	< 0·001
I50 heart failure, n (%)	142, 5·7%	106, 9·4%	< 0·001
E87 Electrolyte disorder, n (%)	36, 1·5%	126, 11·1%	< 0·001
E86 Volume deficit, n (%)	28, 1·1%	92, 8·1%	< 0·001
Triage, n (%)			
Red	116 (4·7)	23 (2·0)	< 0·001
Orange	873 (35·3)	290 (25·6)	< 0·001
Yellow	1281 (51·9)	626 (55·3)	0·056
Green	184 (7·4)	173 (15·3)	< 0·001
Blue	13 (0·5)	16 (1·4)	0·010
Death, n (%)	176 (7·13)	92 (8·13)	0·316

Sensitivity analyses were performed using segmented regression models to compare various temperature metrics (daily mean and maximum temperatures over 1-, 3-, 5-, and 7-day windows). The 5-day mean temperature was identified as the most informative predictor, demonstrating the highest explanatory power (adjusted R² = 0·382) and the most robust risk signal above the identified threshold (t = 3·04) compared to shorter lags (Table 2). This suggests that cumulative thermal exposure over a 5-day window better characterizes the development of diuretic-associated ADRs.

**Table 2:** Comparison of segmented regression models for different mean temperature lag windows, adjusted by demographics and precipitation

Metric	Breakpoint (°C)	Adjusted *R*²	t-Value (Slope 2)
1-day mean temperature	15·4	0·165	1·75
3-day mean temperature	14·7	0·345	2·90
5-day mean temperature	**15·7**	**0·382**	**3·04**
7-day mean temperature	15·0	0·258	2·39
1-day max temperature	22·5	0·092	1·84
3-day max temperature	20·5	0·188	1·98
5-day max temperature	21·0	0·241	2·28
7-day max temperature	21·0	0·160	1·76

**Fig. 1:**
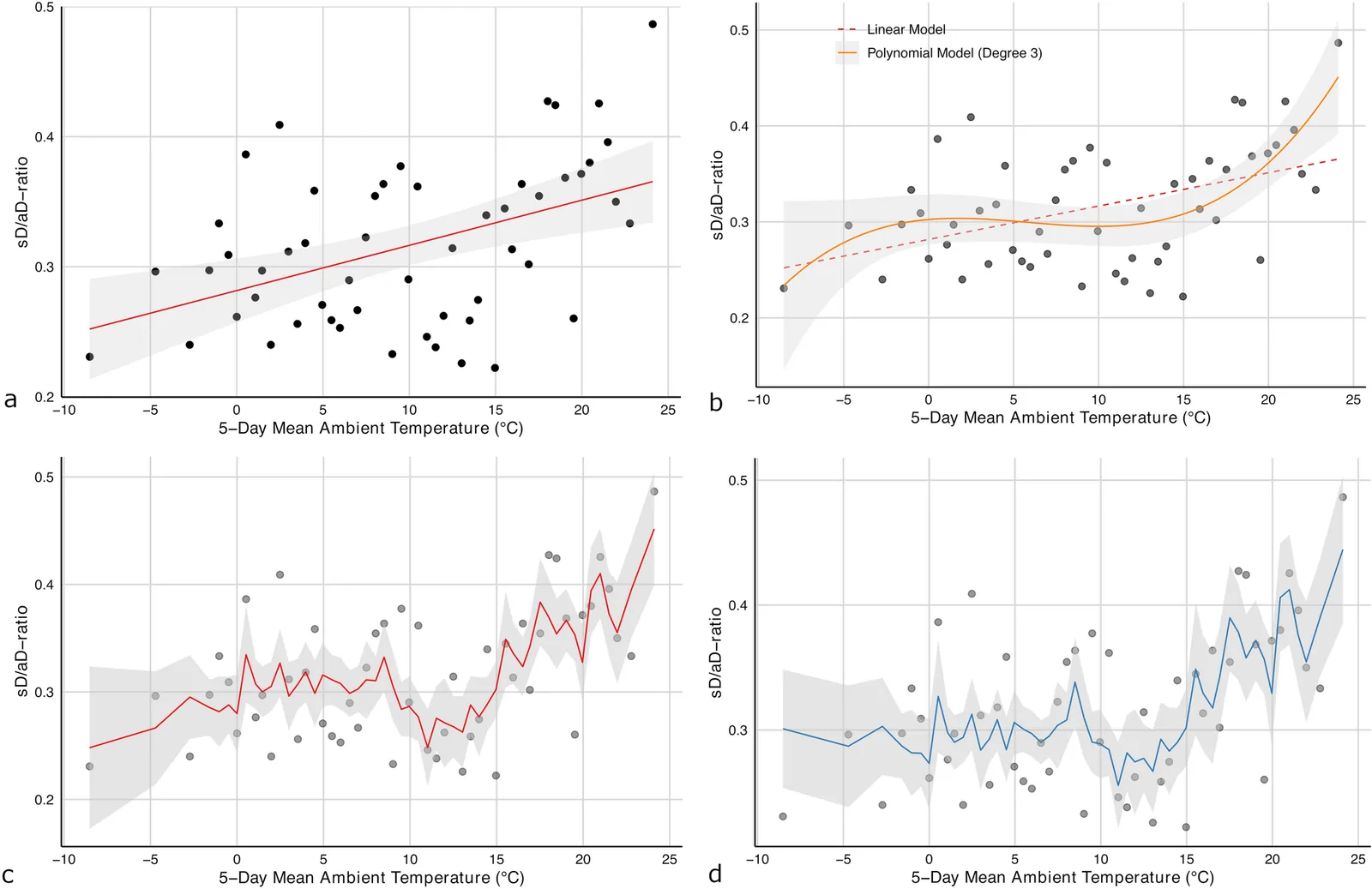
Relationship between 5-day mean temperature and the ratio of suspected diuretic-related adverse drug reactions to all diuretic users (sD/aD-ratio) modeled via different regression approaches. **a** Linear regression line (red solid line) with 95% confidence bands (light gray area). **b** Polynomial regression of 3rd degree (orange solid line) compared to linear model (red dashed line) with 95% confidence bands of the polynomial fit. **c** Natural cubic spline regression (df = 3) highlighting the non-linear acceleration of ADRs at higher temperatures (Adjusted R² = 0·399); line represents marginal effect predictions adjusted for age, sex, and precipitation. **d** Segmented linear regression line (blue solid line) identifying a structural breakpoint at 15·7 °C with 95% confidence bands; line represents marginal effect predictions adjusted for age, sex, and precipitation. All panels plot 5-day mean ambient temperature (°C) on the X-axis and the sD/aD-ratio on the Y-axis

Linear regression showed a significant positive correlation with 5-day mean temperature (β = 0·0035, 95% CI: 0·0015 − 0·0054, *p* < 0·001, adjusted R² = 0·188). The relationship is depicted in Fig. 1a. When adjusted for age, sex, and precipitation, the association with 5-day mean temperature remained significant (β = 0·0037, 95% CI: 0·0010 − 0·0063, *p* = 0·0070), with an overall adjusted R² = 0·179 (Table 3).

**Table 3:** Multivariate linear regression analyzing the relationship between 5-day mean temperature and the ratio of suspected diuretics to all diuretics (sD/aD-ratio) adjusted by demographics and precipitation

Term	Estimate	Std. Error	t-Statistic	*p*-value	95% CI
(Intercept)	0·5545	0·3453	1·6058	0·1149	-0·1398–1·2487
Temperature (in °C)	0·0037	0·0013	2·8170	0·0070	0·0010–0·0063
Age (in years)	-0·0029	0·0046	-0·6363	0·5276	-0·0123–0·0064
Sex (female)	-0·1219	0·1135	-1·0739	0·2883	-0·3502–0·1064
Precipitation (mm)	0·0045	0·0103	0·4346	0·6658	-0·0163–0·0252

We further modeled the relationship using a polynomial regression including linear, quadratic, and cubic terms, adjusted for age, sex, and 5-day mean precipitation. This model was significant overall (F(6, 46) = 6·82, *p* < 0·0001), explaining 40·2% of the variance in the sD/aD-ratio. While the linear term was non-significant (β = 0·1263, 95% CI: -0·0097 − 0·2624, *p* = 0·0680), the quadratic (β = 0·1587, 95% CI: 0·0528-0·2646, *p* = 0·0042) and cubic terms (β = 0·1837, 95% CI: 0·0813-0·2862, *p* = 0·0008) were highly significant. This confirms an accelerating increase in diuretic-associated ADRs as ambient temperature rises, suggesting a critical tipping point in risk (Fig. 1b). Notably, precipitation emerged as a significant independent co-predictor in this non-linear framework (*p* = 0·0157).

### Threshold identification (segmented and spline regression)

To explore whether a threshold effect existed, a segmented linear regression adjusted for age, sex, and precipitation was applied to search for a structural break in the relationship between 5-day mean temperature and sD/aD-ratio (Fig. 1d).

The model identified a breakpoint at 15·7 °C (SE = 1·47). Below the breakpoint, the slope was not significantly different from zero (β = -0·0023, 95% CI: -0·0060 − 0·0014, *p* = 0·215). However, above the breakpoint, the relationship was significantly positive (β = 0·0201, 95% CI: 0·0068 − 0·0333, t = 3·04). The Davies’ test supported the presence of a breakpoint by showing a statistically significant change in slope (*p* = 0·0016). Adding the breakpoint resulted in an improved model fit compared to the multivariate linear model, increasing the explained variance from adjusted R² = 0·179 to adjusted R² = 0·382, which was almost on par with the polynomial model (adjusted R² = 0·402). The inclusion of the weekday of ED admission led to model overfitting without adding predictive value. Results and further discussion can be found in supplement 2.

To capture the complex curvature and potential tipping points without the constraints of global polynomial terms, a natural cubic spline model (df = 3) was fitted (Fig. 1c). This model, also adjusted for age, sex, and precipitation, provided a high statistical fit (adjusted R² = 0·399). While the spline functions for lower temperature ranges showed no significant slope (*p* = 0·1006), the spline basis function corresponding to the highest temperatures was highly significant (*p* < 0·0001). This supports a statistically significant change of slopes, where a period of relative stability of the ratio at lower temperatures is followed by a sharp, non-linear increase in the risk of diuretic-associated ADRs once ambient temperatures exceed moderate levels (Table 4).

**Table 4:** Multivariate natural cubic spline regression (df = 3) results analyzing the relationship between 5-day mean temperature and sD/aD-ratio

Term	Estimate	Std. Error	t-Statistic	*p*-value	95% CI
(Intercept)	0·4815	0·2947	1·6341	0·1091	-0·1116–1·0747
Spline basis 1	-0·0660	0·0394	-1·6754	0·1006	-0·1453–0·0133
Spline basis 2	0·1695	0·0905	1·8731	0·0674	-0·0126–0·3516
Spline basis 3	0·1356	0·0303	4·4706	< 0·0001	0·0746–0·1967
Age (in years)	-0·0026	0·0041	-0·6420	0·5240	-0·0108–0·0056
Sex (female)	-0·1145	0·0972	-1·1773	0·2451	-0·3102–0·0813
Precipitation (mm)	0·0238	0·0099	2·4028	0·0204	0·0039–0·0438

To further isolate the independent impact of temperature from confounding patient demographics and environmental factors, marginal effect predictions were applied to the most robust non-linear models (Fig. 1c and d). By mathematically standardizing age, sex distribution, and precipitation at their cohort means, we were able to generate a risk trajectory for an idealized, averaged patient. The persistence of the non-linear increase after standardization shows that the observed association was not fully explained by measured differences in patient demographics or precipitation.

We applied the AIC (Akaike Information Criterion) to take the increased complexity of the segmented and spline models into account for the comparison of the model performances with the multivariate linear model. The comparison resulted in AIC_linear_ = -147·87, AIC_segmented_ = -161·13 (∆AIC = 13·26), and AIC_spline_ = -162·62 (∆AIC = 14·75), indicating that both non-linear models provided a substantially better fit than the simple linear model.

### Comparison with beta-blocker-associated ADRs

To assess whether the observed thermal sensitivity was specific to diuretics or represented a general increase in ED admissions for any ADR, we performed an identical analysis for beta-blocker-associated ADRs. Sensitivity analyses comparing various temperature metrics identified the 3-day maximum temperature as the most informative predictor for beta-blocker-associated ADRs, though mean temperatures also showed significant associations at various lags (Table 5).

**Table 5:** Comparison of segmented regression models for different temperature metrics and lag windows for beta-blocker-associated ADRs

Metric	Breakpoint (°C)	Adjusted *R*²	t-Value (Slope 2)
1-day mean temperature	13.5	0.197	-1.56
3-day mean temperature	13.2	0.243	-0.98
5-day mean temperature	9.0	0.188	-1.11
7-day mean temperature	8.5	0.173	-0.82
1-day max temperature	11.5	0.254	-1.05
3-day max temperature	20.4	0.271	-2.02
5-day max temperature	11.9	0.237	-1.40
7-day max temperature	13.0	0.183	-0.73

**Fig. 2:**
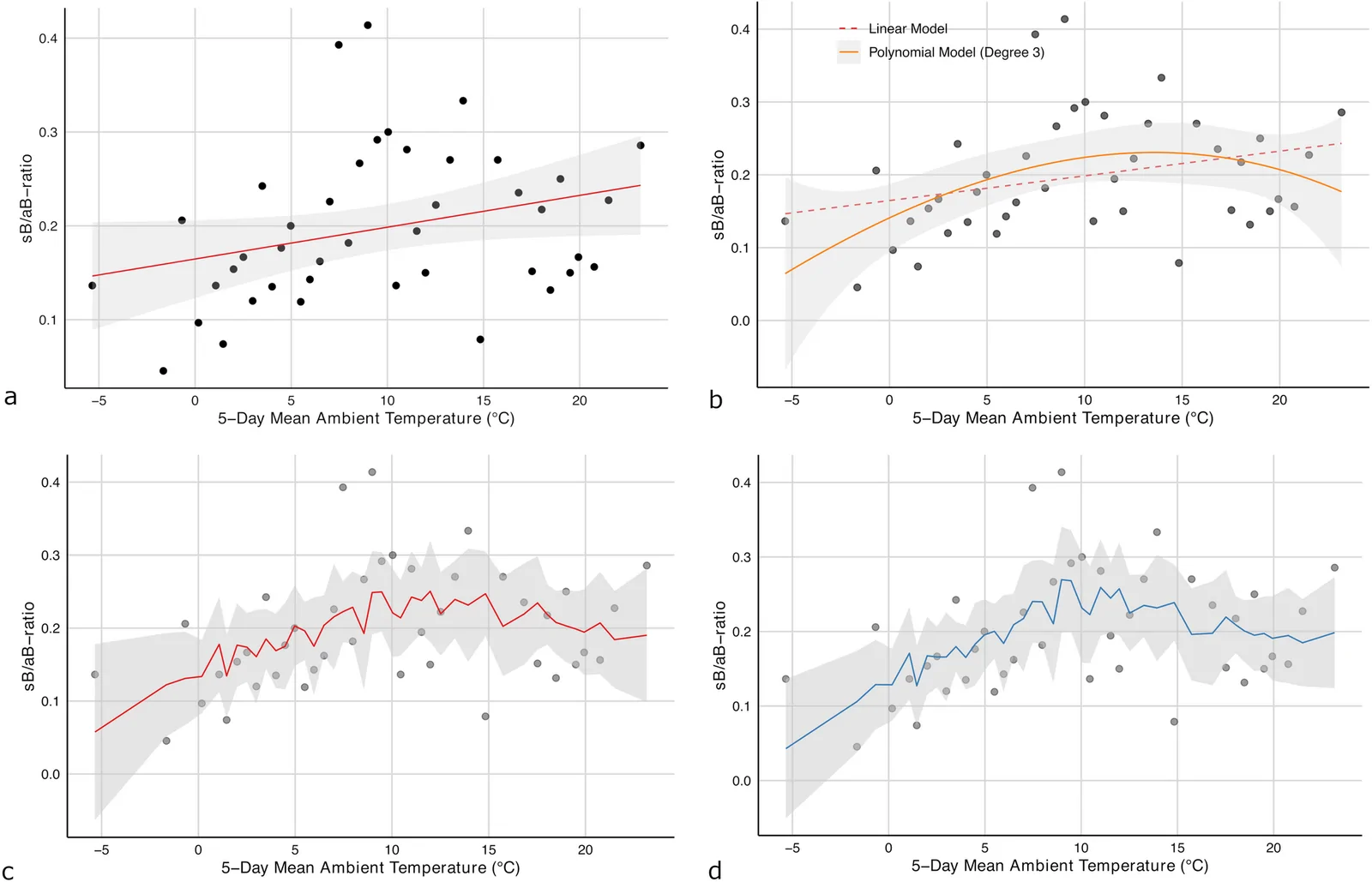
Relationship between 5-day mean temperature and the ratio of suspected beta-blocker-related adverse drug reactions to all beta-blocker users (sB/aB-ratio) modeled via different regression approaches. **a** Linear regression line (red solid line) with 95% confidence bands (light gray area). **b** Polynomial regression of 3rd degree (orange solid line) compared to linear model (red dashed line) with 95% confidence bands of the polynomial fit. **c** Natural cubic spline regression (df = 3) highlighting shifts at lower temperatures (Adjusted R² = 0·1101); line represents marginal effect predictions adjusted for age, sex, and precipitation. **d** Segmented linear regression line (blue solid line) identifying a structural breakpoint at 9·0 °C with 95% confidence bands; line represents marginal effect predictions adjusted for age, sex, and precipitation. All panels plot 5-day mean ambient temperature (°C) on the X-axis and the sB/aB-ratio on the Y-axis

In a multivariate linear model adjusted for age, sex, and precipitation, beta-blocker-associated ADRs showed no significant relationship with 5-day mean temperature (β = 0·0027, 95% CI: -0·0014 − 0·0068, *p* = 0·1879). The explanatory power of this model (adjusted R² = 0·058) was substantially lower than that of the diuretic model (adjusted R² = 0·179; Fig. 2a).

Non-linear modeling for beta-blocker users further highlighted these differences. A 3rd-degree polynomial model, adjusted for age, sex, and precipitation, showed a modest model fit (adjusted R² = 0.089). Within this model, neither the linear term (β = 0.1702, 95% CI: −0.0241–0.3645, *p* = 0.0840), the quadratic term (β = −0.1614, 95% CI: −0.3427-0.0200, *p* = 0.0795) nor the cubic term reached statistical significance (β = −0.0117, 95% CI: −0.1739 − 0.1504, *p* = 0.8840). Similarly, a natural cubic spline model achieved an adjusted R² = 0.110. Of the spline basis functions, only the first basis function was significantly associated with the outcome (Basis 1: β = 0.1493, 95% CI: 0.0145–0.2842, *p* = 0.0309), whereas the second (β = 0.2767, 95% CI: −0.0282–0.5816, *p* = 0.0739) and third basis functions (β = 0.0501, 95% CI: −0.0524–0.1527, *p* = 0.3280) were not statistically significant (Fig. 2c). Notably, neither the whole polynomial nor the whole natural cubic spline model were statistically significant (overall model p-values = 0.159 and 0.119, respectively). Segmented regression identified a structural breakpoint at a lower temperature of 9·0 °C (SE = 2·24). Below this threshold, the risk increased significantly with temperature (β = 0.0137, 95% CI: 0.0048–0.0225, *p* = 0.0034). Above the breakpoint, the association was no longer significant, and the slope became slightly negative (β = −0.0047, 95% CI: −0.0125–0.0031, t = − 1.22; Fig. 2d). Importantly, the Davies’ test for a change in slope was not statistically significant (*p* = 0·0762).

Consistent with the primary analysis, marginal effect predictions were applied to the non-linear beta-blocker models (Fig. 2c and d) to isolate the independent effect of temperature.

## Discussion

Our analyses show a higher prevalence of diuretic-associated ADRs in ED admissions with increasing ambient temperature, with the effect becoming particularly pronounced at 5-day mean temperatures of 15·7 °C and above.

This association between ambient temperature and diuretic-associated ADRs aligns well with existing literature. Diuretic-treated patients are inherently susceptible to heat because of the loss of fluids through excess diuresis, leading to dehydration [16, 17] which is supported by observed admission diagnoses in analyzed ADRs. In addition, patients taking these medications often suffer from underlying cardiovascular or renal diseases [16], posing an additional vulnerability to fluid loss, which could also be observed in our data set.

While daily maximum temperature also showed significant associations, its predictive power was consistently lower than that of the 5-day mean temperature, suggesting that 5-day mean temperature better capture the cumulative thermal exposure relevant to developing a diuretic-associated ADR. Sensitivity analyses across various lags (1-day to 7-day) demonstrated that the 5-day window provided the most robust statistical fit (adjusted R² = 0·382), whereas single-day metrics lacked comparable informational value. This suggests that mortality and morbidity risk - specifically diuretic-related ADRs - may disproportionately rise with consecutive days of heat stress rather than isolated peaks. Previous research has similarly linked daily mean temperature to mortality [18]. The explanation why the mean temperature is more informative than the maximum temperature could be that the nocturnal temperature may stem a critical role in the physical recovery from the daytime heat [19] and the combination of high nighttime and daytime temperature imposes a greater risk than isolated daytime peaks [20]. Meteorologically, cumulative values are often preferred over single-day values, as seen in the definition of the heat wave, the Nairn-Fawcett Extreme Heat Factor [21], or the Excess Heat Factor [22], that measures the severity of a heat wave. Epidemiologically, a 1- to 3-day lag in heat-related health effects has been established, on which the definition of the Excess Heat Factor is based [22]. Single-day measurements may lack the informational value as mortality risk rises disproportionately with consecutive days of heat [21]. It is therefore plausible that multiple consecutive days of heat must also be considered when analyzing the side effects of diuretics.

The significance of the quadratic and cubic terms in the polynomial model, along with the segmented and spline regressions, confirms that the relationship between temperature and sD/aD-ratio is not purely linear. The AIC comparison, showing a substantial improvement from AIC_linear_ (-147·87) to AIC_spline_ (-162·62), further supports the use of non-linear frameworks. This finding is biologically plausible, as no specific diuretic-associated ADRs are expected at cold temperatures. While our segmented regression showed no statistically significant association between temperature and the sD/aD-ratio below the 15·7 °C breakpoint, this does not necessarily demonstrate the complete absence of an effect at lower temperatures. Rather, it indicates that the risk signal becomes dominant and clinically observable only after this breakpoint is crossed, at which stage temperature is sharply and positively associated with the outcome.

The identified breakpoint of ~ 15·7 °C aligns with the established mortality-temperature curves. Previous research showed similar non-linear relationships between mortality and temperature [23]. This study identified a global mortality minimum around the mean temperature 15–20 °C, after which a seemingly polynomial increase could be observed as the temperature rises. Other research confirms a temperature optimum with lowest mortality rates around 17–19 °C [24]. However, it has also been shown that populations in different locations are sensitive to different temperature thresholds, which is why the described optimum temperature range must be considered with caution in respect of time and location [25]. In Germany, a significant increase in mortality was observed starting at a weekly mean temperature of 20 °C [26]. While Achebak et al. examine the mortality of several different diseases, we focus on potential ADRs of a single drug group primarily associated with heat vulnerability [16]. Since mortality describes an extreme outcome that normally occurs after symptom progression, we assume that the breakpoint must be located at a lower temperature than the mortality curve minimum. The rising temperature then initially would lead to an increase in symptoms - in our case, the ADRs attributed to diuretics - before a rise in mortality can finally occur. While Achebak et al. focus on the general population, our cohort is characterized by advanced age, polypharmacy and chronic pre-existing conditions, which likely explains this stronger sensitivity to even moderate temperature increases, thereby explaining a lower breakpoint.

As an exploratory active-comparator analysis, we applied the same modeling strategy to suspected beta-blocker-associated ADRs. While our models detected a nominal heat-related signal for beta-blocker users for lower temperatures, the association was weak and often non-significant. Furthermore, the identified breakpoint for diuretics (~ 15·7 °C) was relatively consistent across different sensitivity analyses and modeling approaches. In contrast, the breakpoint for the beta-blocker group (~ 9·0 °C) was highly inconsistent and lacked robust statistical support (Davies’ test, *p* = 0·0762), with the side-effect ratio reaching a plateau early in the temperature range. Because the models were fitted separately, these findings should be interpreted as a descriptive comparison of the two-temperature-response patterns. While diuretics exhibit a statistically significant response to thermal stress, it remains questionable whether beta-blocker-associated ADRs demonstrate a meaningful temperature-related effect in this population.

Marginal-effect predictions allowed the temperature association to be estimated while holding age, sex, and precipitation constant. The persistence of the non-linear pattern after adjustment suggests that the temperature-ADR association was not determined by these measured covariates and may instead reflect an association with cumulative thermal stress. This finding also shows that the estimated breakpoint at approximately 15·7 °C stayed stable after adjustment for the included demographic and meteorological covariates.

Furthermore, the robustness of this signal is underscored by our supplemental analysis adjusting for the day of the week. The inclusion of day of the week variables, while accounting for potential temporal admission trends, failed to yield significant coefficients and degraded the model’s adjusted explanatory power. These statistical data indicate that temperature might be the dominant and independent driver of the observed risk increase, whereas temporal factors such as weekly cycles are negligible in comparison.

There are limitations to the analysis. Our underlying data set consists of patients who have all experienced ADRs from different medications. The calculated symptom-drug ratio compares patients experiencing diuretic-associated ADRs to those with other ADRs, potentially creating numerical shifts in temperature analysis. For example, commonly prescribed non-diuretic medications could also show a strong temperature-related ADR and therefore decrease the sD/aD-ratio, leading to a false negative or false absent association between temperature and diuretics. While also other drugs, such as ACE inhibitors may expose patients to a heat-related risk [27, 28], our primary analysis solely focuses on diuretics and uses beta-blockers as an active comparator. Nevertheless, beta-blocker users do not represent a general non-diuretic population and may differ from diuretic users. Furthermore, the temperature-response patterns were estimated in separate models and compared descriptively. Future studies should examine additional drug groups and use combined models that formally test interactions between medication groups and temperature. Our cohort’s overall vulnerability - characterized by advanced age and polypharmacy - may increase susceptibility to extreme temperatures, potentially explaining our relatively low 15·7 °C threshold. However, this represents a clinically relevant cohort for the occurrence of ADRs. Finally, limited data availability at extreme temperatures required pooling adjacent temperature steps, which reduced the number of data points for regression analysis and may have influenced the exact estimated threshold and curve shape. However, this predefined grouping strategy was chosen to avoid unstable ratio estimates in sparsely populated temperature ranges.

## Conclusions

In conclusion, this study shows a significant, non-linear relationship between ambient temperature and the occurrence of diuretic-associated ADRs. The strongest effect could be observed for the 5-day mean temperature, with a significant threshold starting around 15·7 °C. These findings suggest that even moderate heat may increase ADR risk in a vulnerable population and emphasize the need to incorporate environment factors including ambient temperature into clinical risk assessments for patients on diuretics.

## Supplementary Information

Below is the link to the electronic supplementary material.

Supplementary Material 1

## Data Availability

The individual-level patient data analyzed during the current study are not publicly available due to ethical and data protection regulations. Data are available from the corresponding author upon reasonable request and with permission of the responsible ethics committee. All analysis codes are publicly available in the following GitHub repository: https://github.com/Fledermaus12/ADREDWeather. Access requests will be assessed within 8 weeks and require a data use agreement specifying restrictions on data handling and reuse.

## Abbreviations

ADR: adverse drug reaction
AIC: Akaike Information Criterion
ED: emergency department
AKI: acute kidney injury
CKD: chronic kidney injury
ATC: Anatomical Therapeutic Chemicals
sD/aD-ratio: ratio of suspected diuretics to all taken diuretics
