# Supplementary material for: Diuretic-associated admissions to the emergency department – too hot to handle? Results from the ADRED-study
Source: BMC Med. 2026 Nov 7;24:633. doi: 10.1186/s12916-026-05370-1 (PMC13757121; doi:10.1186/s12916-026-05370-1)
Supplement: Supplementary file 1 [file 12916_2026_5370_MOESM1_ESM.docx]

**Supplements**

**Supplement 1: Overview of diuretics and their ATC-codes used in study**

| **Drug(group)** | **Subgroup** | **ATC-code** |
| --- | --- | --- |
| Thiazides |  | C03A |
|  | Thiazides in combination with potassium-sparing agents | C03EA |
|  | ACE inhibitors and diuretics | C09BA |
|  | ARBs and diuretics | C09DA |
|  | Beta blocking agents and thiazides | C07B |
| Other low-ceiling diuretics | Other low-ceiling diuretics, plain | C03BA |
|  | Beta blocking agents and other diuretics | C07C |
| Loop diuretics | Loop diuretics, plain | C03C |
|  | Loop diuretics and aldosterone antagonists | C03ED |
| Aldosterone antagonists and potassium-sparing agents |  | C03D |
|  | Loop diuretics and aldosterone antagonists | C03ED |

**Supplement 2: The inclusion of day of weeks of ED admission as coefficients in segmented regression.**

To address potential temporal trends in ED admissions, an additional analysis was performed for the segmented regression by further adjusting for the day of the week. This model identified a breakpoint at 16·9 °C (SE = 1·22), with no significant slope before the threshold (β = -0·0023, 95% CI: -0·0062 - 0·0016, p = 0·243) and a significant positive slope thereafter (β = 0·0270, 95% CI: 0·0077 - 0·0464, t = 2·8321). While the Multiple R² increased to 0·507, the Adjusted R² decreased to 0·343 (from 0·382 in the primary adjusted model), and all individual days of the week variables were statistically non-significant (all p > 0·17). This indicated that the inclusion of day of the week variables led to model overfitting without adding predictive value, thereby supporting the exclusion of these variables in the primary analysis for greater statistical efficiency.
